# Estimating Weekly National Opioid Overdose Deaths in Near Real Time Using Multiple Proxy Data Sources

**DOI:** 10.1001/jamanetworkopen.2022.23033

**Published:** 2022-07-21

**Authors:** Steven A. Sumner, Daniel Bowen, Kristin Holland, Marissa L. Zwald, Alana Vivolo-Kantor, Gery P. Guy, William J. Heuett, DeMia P. Pressley, Christopher M. Jones

**Affiliations:** 1 National Center for Injury Prevention and Control, US Centers for Disease Control and Prevention, Atlanta, Georgia; 2 Division of Violence Prevention, US Centers for Disease Control and Prevention, Atlanta, Georgia; 3 Division of Overdose Prevention, US Centers for Disease Control and Prevention, Atlanta, Georgia; 4 Diversion Control Division, US Drug Enforcement Administration, Springfield, Virginia

## Abstract

**Importance:**

Opioid overdose is a leading public health problem in the United States; however, national data on overdose deaths are delayed by several months or more.

**Objectives:**

To build and validate a statistical model for estimating national opioid overdose deaths in near real time.

**Design, Setting, and Participants:**

In this cross-sectional study, signals from 5 overdose-related, proxy data sources encompassing health, law enforcement, and online data from 2014 to 2019 in the US were combined using a LASSO (least absolute shrinkage and selection operator) regression model, and weekly predictions of opioid overdose deaths were made for 2018 and 2019 to validate model performance. Results were also compared with those from a baseline SARIMA (seasonal autoregressive integrated moving average) model, one of the most used approaches to forecasting injury mortality.

**Exposures:**

Time series data from 2014 to 2019 on emergency department visits for opioid overdose from the National Syndromic Surveillance Program, data on the volume of heroin and synthetic opioids circulating in illicit markets via the National Forensic Laboratory Information System, data on the search volume for heroin and synthetic opioids on Google, and data on post volume on heroin and synthetic opioids on Twitter and Reddit were used to train and validate prediction models of opioid overdose deaths.

**Main Outcomes and Measures:**

Model-based predictions of weekly opioid overdose deaths in the United States were made for 2018 and 2019 and compared with actual observed opioid overdose deaths from the National Vital Statistics System.

**Results:**

Statistical models using the 5 real-time proxy data sources estimated the national opioid overdose death rate for 2018 and 2019 with an error of 1.01% and −1.05%, respectively. When considering the accuracy of weekly predictions, the machine learning–based approach possessed a mean error in its weekly estimates (root mean squared error) of 60.3 overdose deaths for 2018 (compared with 310.2 overdose deaths for the SARIMA model) and 67.2 overdose deaths for 2019 (compared with 83.3 overdose deaths for the SARIMA model).

**Conclusions and Relevance:**

Results of this serial cross-sectional study suggest that proxy administrative data sources can be used to estimate national opioid overdose mortality trends to provide a more timely understanding of this public health problem.

## Key Points

**Question:**

Can proxy data sources be used to estimate national opioid overdose mortality trends in near real time?

**Finding:**

In this cross-sectional time series analysis, signals from 5 overdose-related, proxy data sources encompassing health, law enforcement, and online data from 2014 to 2019 in the US were combined via a statistical model that was able to demonstrate that these data can be used to estimate national opioid overdose death rates with an approximate 1% error.

**Meaning:**

This study suggests that it may be possible to enable a more timely understanding of national opioid overdose mortality trends through the use of near–real-time proxy data sources.

## Teaser

This cross-sectional study uses time series data to build and validate a statistical model for estimating national opioid overdose deaths in near real time.

## Introduction

Opioid overdose is a leading public health problem in the United States, with opioids involved in approximately 50 000 deaths in 2019.^1^ Rapid changes in substance use patterns, the emergence of highly potent synthetic opioids and other illicit drugs associated with an increased risk of overdose, and external events, such as the COVID-19 pandemic, have challenged prevention efforts.^2,3,4^ Timely resource allocation and response are necessary to preventing further escalation of overdose deaths.

However, anticipating shifts in national epidemiologic trends associated with the opioid overdose crisis is difficult. Mortality data derived from death certificates are reported by the Centers for Disease Control and Prevention (CDC) to provide official counts of overdose deaths involving opioids and other drugs in the United States. However, such information is compiled from local coroners’ and/or medical examiners’ offices and state vital records offices nationwide and have historically lagged 1 or more years owing to challenges ranging from increased time needed to conduct postmortem toxicologic testing to limited information technology infrastructure in some jurisdictions.^1,5^

To address these challenges, the CDC has begun releasing provisional mortality estimates based on preliminary records received; however, such estimates are still delayed by approximately 6 months.^6^ Although infectious disease modeling communities have done considerable work to advance forecasting methods for health topics such as influenza monitoring, for which data are delayed by approximately 1 to 2 weeks,^7^ there is limited work in national opioid overdose forecasting.

Nonetheless, while mortality data on opioid overdose continue to be delayed, there are multiple data sources that are available in real time or with minimal delay that can potentially provide insights into emerging overdose mortality trends. Such data sources include syndromic surveillance data from emergency departments (EDs), law enforcement–derived data, and online data sources.^8,9,10,11,12,13^ Consequently, we hypothesized that contemporary machine learning approaches^14^ designed to combine information from multiple data streams could be used to provide more timely estimates of national opioid-involved overdose deaths.

## Methods

### Data Sources

To address the lack of real-time estimates of opioid-involved overdose deaths in the United States, we developed and validated a modeling approach known as “nowcasting.”^15^ In general, nowcasting approaches attempt to use proxy data sources that are available in near real time to impute or estimate trends in an outcome lacking real-time data (in our case, opioid-involved overdose deaths). Consequently, we identified 5 data sources with theoretically justifiable associations with opioid-involved overdose death trends that are available in real time or near real time. This work constituted secondary analysis of deidentified data and was therefore exempt from Centers for Disease Control and Prevention institutional review board review and adheres to the Strengthening the Reporting of Observational Studies in Epidemiology (STROBE) reporting guideline for cross-sectional studies. No informed consent was required.

The first category of data sources represent data from official health-related and drug supply sources. This category includes national ED visit trends for opioid overdose from the CDC’s National Syndromic Surveillance Program.^8^ More than 71% of ED facilities in 49 states and the District of Columbia share data with the National Syndromic Surveillance Program.^16^ Opioid overdose ED visits were identified using standardized discharge diagnosis codes and “chief complaint” text fields.^17,18,19^ For our modeling, we used the daily rate of ED visits for opioid overdose, defined as the daily count of opioid overdose visits divided by the total number of daily ED visits for any reason. The second data source that we used in this category was data from the National Forensic Laboratory Information System (NFLIS-Drug), which is a US Drug Enforcement Administration program.^20^ The NFLIS-Drug maintains a network of federal, state, and local forensic laboratories across the US that analyze and report identifications of substances. This system provides timely information on which drugs may be circulating in drug markets and causing harm. From NFLIS-Drug, we tabulated daily counts of heroin and synthetic opioids (ie, fentanyl, carfentanil, and furanylfentanyl) submitted to the US Drug Enforcement Administration. We focused on heroin and synthetic opioids because these are the primary substances associated with opioid-involved overdose deaths in the US.^21^

The second category of data that we used represents data from online sources. Such data have been widely used in published research to understand substance use trends.^10,11,22^ First, we included Google search trends data, which are publicly available and are derived from key word searches performed on the Google search engine.^23,24^ From Google Trends, we queried weekly search trends over a rolling 5-year period for heroin and common synthetic opioids (heroin, fentanyl, carfentanil, acrylfentanyl, furanylfentanyl, and acetylfentanyl). For each drug term, Google Trends returned a normalized value from 0 to 100, indicating the relative popularity of that search term over the time period specified (with increasing values indicating increasing popularity). Consistent with prior research,^14^ we summed the values for each of the key words into a single weekly score. Second, we included weekly trend information on posts related to heroin and synthetic opioids from the social media platforms Twitter and Reddit. Twitter is a news-focused social media platform, and research using Twitter has revealed a moderate correlation with substance use health indicators derived from traditional data sources.^10^ Reddit is a forum-based social media platform allowing for longer-form question and answer exchanges and has become a leading global site for communities of people who use drugs and those seeking recovery to discuss their experiences.^12,13,25^ Data from each platform represented the total weekly count of all posts mentioning a heroin-related or synthetic opioid–related key word. This information was queried using a commercial communications platform with full indexing of all Twitter and Reddit content.^26^

### Outcome Variable

The aforementioned 5 data streams were used to estimate weekly opioid-involved overdose deaths as the primary outcome variable in our study. The temporal unit of 1 week was chosen because it represented a time interval that was considered useful for public health practice and also enabled a sufficient amount of training data for the modeling process (approximately 52 data points per year). Weekly counts of opioid-involved overdose deaths were derived from analysis of death certificate data from the CDC’s National Vital Statistics System possessing the *International Statistical Classification of Diseases and Related Health Problems, Tenth Revision* (*ICD-10*) underlying cause of death codes X40 to X44 (unintentional), X60 to X64 (suicide), X85 (homicide), or Y10 to Y14 (undetermined intent) as well as multiple cause of death *ICD-10* codes specific to the opioid drug category: code T40.0 (opium), code T40.1 (heroin), code T40.2 (natural and semisynthetic opioids), code T40.3 (methadone), code T40.4 (synthetic opioids other than methadone), or code T40.6 (other and unspecified narcotics). For the development and evaluation of our modeling approach, we calculated opioid-involved overdose deaths from 2010 through 2019, the most recent year for which official final mortality data were available from the CDC. We validated our prediction models separately for both 2018 and 2019 to develop an approach that was robust across multiple years. Validating our approach for both 2018 and 2019 presented an ideal challenge because significant shifts in opioid-involved overdose deaths occurred during this time period. After a multidecade increase in opioid-involved overdose deaths, 2018 saw the first decrease in opioid overdose deaths, followed by a reversal of this decrease and a subsequent increase in deaths in 2019.

### Statistical Analysis

We included data from each of the 5 data sources in a LASSO (least absolute shrinkage and selection operator) regression model to predict weekly opioid-involved overdose death counts.^27^ LASSO models are a form of linear regression that impose an additional penalty term in the model fitting process. We selected LASSO regression for several reasons: LASSO models are frequently tested and used in time series prediction tasks; they can help prevent overfitting in the setting of models with many temporally lagged predictors; and although they are a machine learning model that involves parameter optimization as learned through the training data set, they are also among the most easily interpretable machine learning models because coefficients from these models can be inspected, which is beneficial for public health practice.^28,29^ We built and tested LASSO models incorporating up to 16 weeks of lagged data for each of the 5 data sources. Thus, the largest models had 80 predictor variables, and LASSO models were used to help prevent overfitting. The percentage error in estimating the national opioid overdose fatality rate for varying amounts of lagged data is shown and discussed in the eAppendix in the Supplement. Because model performance and percentage errors were stable (approximate ≤1% error) when using approximately 2 months or less of lagged data (eFigure in the Supplement), we display and discuss results for the most parsimonious model, which uses a given week’s values for the 5 predictor variables to model the same week’s count of overdose deaths. We also conducted a sensitivity analysis using ordinary least-squares regression without any model parameter tuning (eAppendix in the Supplement).

Consistent with contemporary machine learning frameworks, we divided our data set into independent segments for the model training and testing process. As noted, data from 2018 and 2019 were set aside for model testing and not used in any model training to allow for a rigorous assessment of model performance. For each year in which predictions were made, we used data for model training that had a gap of 1 year or more to simulate existing constraints. For example, to predict the count of deaths in each week of 2018, we trained models on data from 2014 through 2016. A 3-year length for our training data was the maximum period that we could use, given the historical availability of all data sources. The optimal parameters for the LASSO model were determined by minimizing the root mean squared error on the training data using a 5-fold cross-validation. LASSO models were fit in Python, version 3.7 (Python Software Foundation) via the widely used *scikit-learn* library.^30^

In addition to the LASSO model, we compared our results with those from a baseline model representing one of the most used approaches to forecasting injury mortality.^31^ Specifically, we used a seasonal autoregressive integrated moving average (SARIMA) time series model; this approach learns from historic mortality data to forecast future trends and has been widely used in leading publications forecasting overdose mortality and other related outcomes.^31^ Similar to the LASSO model, the SARIMA model was built to simulate existing constraints and data availability and thereby included a 1-year gap between training data and test data. To predict 2018 weekly death counts, weekly counts of historic deaths from 2010 through 2016 were used in model training; to predict 2019 weekly death data, weekly counts of historic deaths from 2010 through 2017 were used in model training. SARIMA models were fit in R, version 4.1.2 (R Group for Statistical Computing) using the *forecast* package.^32^ All *P* values were from 2-sided tests and results were deemed statistically significant at *P* < .05.

After training the LASSO models on historical data, we made predictions for weekly opioid-involved overdose deaths for each week in 2018 and 2019. These predictions essentially represent replication of a prospective test of how such models would have performed using only the data available to them at that time. We compared the predicted counts of weekly opioid-involved overdose deaths with the actual counts. Our main metric was the annual error rate; this metric assesses the percentage error in the predicted rate of opioid-involved overdose deaths in the United States if we had relied on our prediction models for death estimates, compared with the actual opioid-involved overdose death rate that year. In addition, we report the Pearson correlation coefficient between predicted and actual deaths, the root mean squared error (the mean number of deaths that predictions deviated from actual values in a given week), and the mean absolute percentage error (the mean percentage error for a given week when comparing predicted with actual values).

## Results

**Figure 1.**
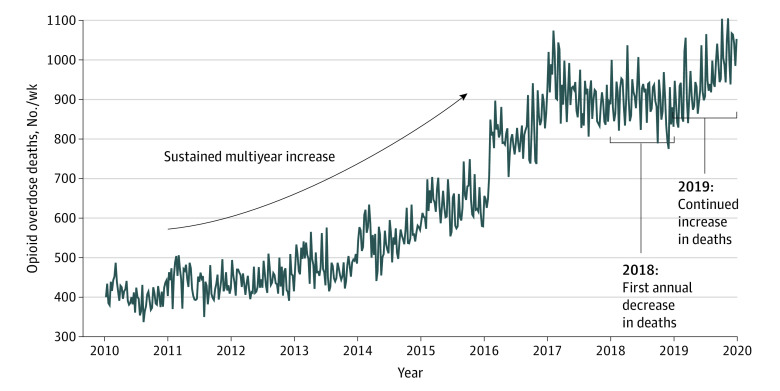
Weekly Number of Drug Overdose Deaths Involving Opioids—National Vital Statistics System, US, 2010-2019 Deaths were classified using *International Statistical Classification of Diseases and Related Health Problems, Tenth Revision* codes. Drug overdose deaths were identified using underlying cause of death codes X40 to X44 (unintentional), codes X60 to X64 (suicide), code X85 (homicide), or codes Y10 to Y14 (undetermined intent), as well as multiple cause of death codes specific to the opioid drug category: T40.0 (opium), T40.1 (heroin), T40.2 (natural and semisynthetic opioids), T40.3 (methadone), T40.4 (synthetic opioids other than methadone), or T40.6 (other and unspecified narcotics).

Figure 1 displays the trend in opioid-involved overdose deaths in the United States from 2010 through 2019. After opioid-involved overdose deaths increased each year from approximately 400 weekly deaths in 2010 to more than 900 weekly deaths in 2017, the first annual decrease in opioid-involved deaths occurred in 2018. This decrease was subsequently followed by a reversal of this trend, with 2019 experiencing a marked increase in overdose deaths.

**Figure 2.**
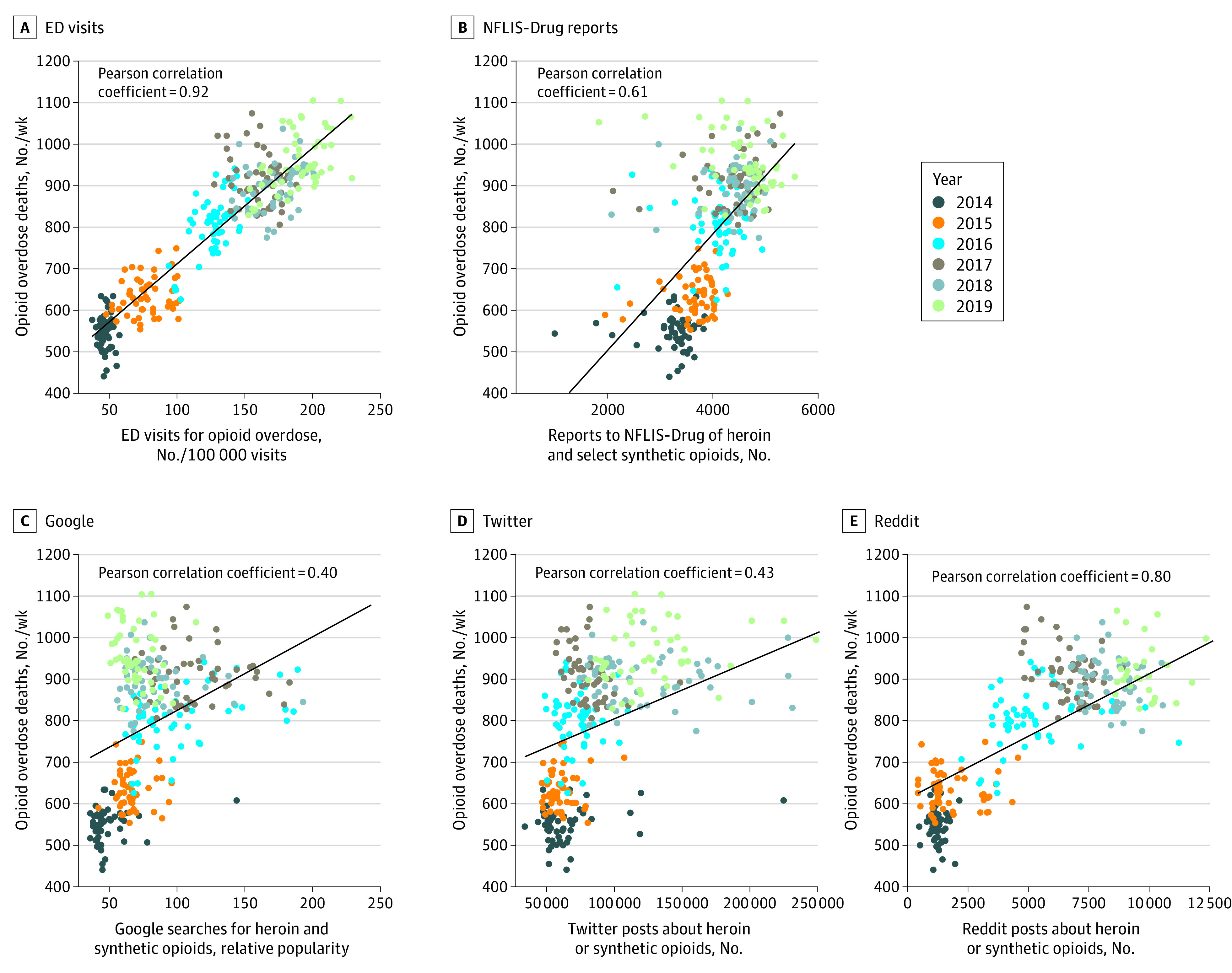
Correlation of Health, Drug Supply, and Online Data Sources With Opioid Overdose Deaths A, Data from the National Syndromic Surveillance Program on emergency department (ED) visits. B, Data from the National Forensic Laboratory Information System (NFLIS-Drug). C, Data from Google Trends. D, Data from Twitter posts. E, Data from Reddit posts.

Figure 2 displays a descriptive scatterplot of each of the potential predictor data sources for nowcasting of deaths. Pearson correlations between each predictor variable and opioid-involved overdose deaths ranged from a low of 0.40 for Google Trends to a high of 0.92 for the ED visit data. All Pearson correlations were statistically significant at the *P* < .01 level.

The Table provides the performance metrics of our LASSO model and the SARIMA model baseline approach to estimate opioid-involved overdose deaths. When predicting overdose deaths for 2018 (the year for which the first decrease in overdose deaths occurred after a multiyear increase), the baseline SARIMA model markedly overestimated the national opioid-involved overdose death rate by 32.8%. For 2019, the SARIMA baseline model showed improved performance but underestimated the death rate by 4.1%.

**Table.** Performance of Models Estimating Opioid-Involved Overdose Deaths for 2018 and 2019, United States^a^

Year and model	Predicted annual rate per 100 000 population	Annual rate error (%)	Pearson correlation	Root mean squared error	Mean absolute % error
2018					
Baseline model	19.0	32.8	−0.203	310.2	33.8
Health and drug supply	14.7	2.9	0.333	67.0	6.1
Online	13.4	−6.7	−0.253	95.7	8.8
Composite (health and drug supply plus online data)	14.5	1.0	0.303	60.3	5.4
2019					
Baseline model	14.6	−4.1	−0.191	83.3	6.6
Health and drug supply	15.4	1.3	0.420	69.7	6.0
Online	16.4	8.2	0.440	140.2	11.1
Composite (health and drug supply plus online data)	15.0	−1.1	0.417	67.2	5.6

^a^ Health and drug supply data sources include emergency department visits from the National Syndromic Surveillance Program and substance identification reports submitted to the National Forensic Laboratory Information System (NFLIS-Drug). Online data sources include Google, Twitter, and Reddit data. The baseline model consists of a seasonal autoregressive integrated moving average (SARIMA) time series model built using historical mortality data.

Conversely, the machine learning–based nowcasting model demonstrated improved performance for both 2018 and 2019. For 2018, the machine learning–based models estimated the annual opioid-involved overdose death rate with 1.0% error compared with the actual rate observed (Table). For 2019, the machine learning–based model had a −1.1% error when estimating the annual opioid-involved overdose death rate. When considering the accuracy of weekly predictions, the machine learning–based approach possessed a mean error in its weekly estimates (root mean squared error) of 60.3 overdose deaths for 2018 (compared with 310.2 overdose deaths for the SARIMA model) and 67.2 overdose deaths for 2019 (compared with 83.3 overdose deaths for the SARIMA model).

**Figure 3.**
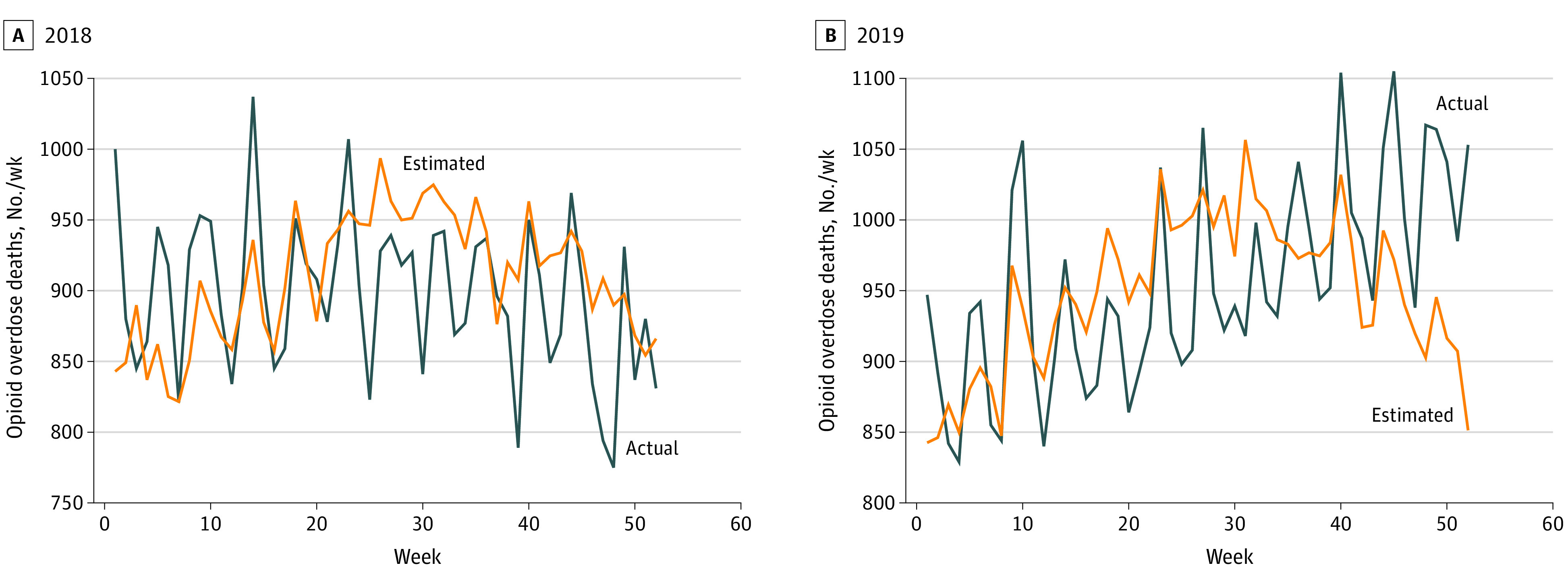
Weekly Opioid-Involved Overdose Deaths From Prediction Model Compared With Observed Deaths, US, 2018-2019 Data sources used in the prediction model include National Syndromic Surveillance Program emergency department data, National Forensic Laboratory Information System reports, Google search trends, Twitter posts, and Reddit posts.

Figure 3 provides a visual depiction of the predictions made by the machine learning–based approach for both 2018 and 2019. Weekly prediction results for 2018 reveal a 0.303 Pearson correlation with observed values. Predictions for 2019 reveal a 0.417 Pearson correlation with observed values, although the model underestimated deaths in the final weeks of 2019.

Subgroup analyses presented in the Table demonstrate that the health and drug supply data sources outperformed the online data sources in their predictive performance. Nonetheless, using all 5 data sources yielded superior estimates compared with using health and drug supply data sources alone.

## Discussion

In this cross-sectional study, we worked to develop and evaluate an approach to help address a longstanding challenge—the lack of near–real-time overdose death estimates. We used a nowcasting-based approach that combined signals from multiple proxy, near–real-time data sources via a machine learning model to estimate weekly opioid-involved overdose deaths in the United States. We validated the modeling approach over 2 separate years that had substantially different overdose death rate trends, and we observed that our nowcasting models yielded promising results, with approximately a 1% error in estimating opioid-involved overdose deaths over an entire year.

Many public health challenges face a lack of timely data, although data delays for injury-related causes of death are particularly severe.^5^ Causes of death from injury can take considerable time to certify, owing to the additional need for laboratory or toxicologic testing and the time required for investigation and determination of cause and manner of death, such as overdose, suicide, or violence.^5^

Classical time series modeling approaches, such as ARIMA (autoregressive integrated moving average)–based models, can encounter challenges when modeling nonstationary series. Although such models are excellent at learning from historical mortality trends, many causes of injury—such as opioid overdose—have experienced multiyear or even multidecade increases.^33^ Thus, models that learn only from observing trends in such historic data have difficulty in predicting rapid and unexpected trend shifts, such as the shift that occurred in 2018 when the first annual decrease in opioid-involved overdose deaths was observed after a period of multiyear exponential growth.^33^ Expectedly, our baseline SARIMA models markedly overestimated deaths for 2018.

### Limitations

Proxy data sources, such as those used in this project, can have notable limitations. Among all data sources, ED visits for overdose had the strongest correlation with overdose deaths, and the coefficients in the LASSO model for the ED visit data were several-fold higher than the other data sources, indicating that this data source had the most predictive power. This finding is intuitive because an ED visit represents a severe clinical outcome, whereas the other data sources are more upstream indicators for overdose (eg, markers of drug supply via NFLIS-Drug data or interest in substances via Google Trends). Greater attention to advancing the use of novel surveillance systems based on electronic health records should be an important aim for public health. The US Drug Enforcement Administration NFLIS-Drug drug-seizure data have been used in prior epidemiologic research^9^ but rely on data from law enforcement and are analyzed by participating laboratories. Nonetheless, it is reasonable to postulate that an understanding of what substances are circulating in the illicit marketplace might help estimate mortality trends because certain substances, such as illicitly manufactured synthetic opioids, are considerably more lethal than other substances.

The online data that we used have also been widely used in previous research given their public nature and accessibility; however, concerns have been raised about the generalizability of these data sources because users of these platforms may not be representative of the entire population and the underlying nature of the administrative data may evolve.^34^ We found that the predictive performance of the online data sources was considerably lower than the health data sources in estimating weekly opioid-involved overdose deaths. Among all the online data sources considered, Google Trends data possessed the largest weight in the LASSO models. The widespread use of internet browsing and the anonymity afforded in internet searches may allow for a truer representation of the population-level interest and demand for substances when compared with specific social media platforms with a more narrow user base. However, prior research has indicated that it is still prudent to attempt to combine information from these alternative signals with traditional sources to improve both performance and reliability of the model.^14^

Although this approach to predicting opioid overdose deaths demonstrated promise, much additional work remains. Although we validated our approach over 2 separate years, future prospective validation is an ongoing need. This work focused on generating mortality estimates at the national level but does not address the need for state or local estimates. Although it is true that some local jurisdictions possess more up-to-date information on epidemiologic trends for overdose deaths in their community, there is considerable variability across the US. There are more than 2000 local medical examiner and coroner offices in the US that are responsible for certifying deaths and generating the data needed to assess overdose mortality trends.^35^ With this considerable decentralization, there is large variability in the timeliness of data by jurisdiction. Even in locales with adequate staffing and modernized systems, speed of reporting is still limited by the postmortem toxicology testing and other components of medicolegal death investigations needed to certify a death as an overdose, which can take several months. Additional work is needed to evaluate the utility of additional data sources and, where local-level data are available, test other modeling approaches, such as those that may incorporate network or spatial information.^36^

## Conclusions

Lack of timely mortality data poses a major challenge for public health efforts. Near–real-time mortality estimates are needed at the national level to help guide federal planning and funding for opioid overdose treatment and prevention. Although the CDC continues to invest in modernization of the underlying public health data infrastructure in the US, use of novel data sources and modeling approaches are also becoming central to public health practice, as underscored during the COVID-19 pandemic. Delayed data result in a reactionary approach to deployment of resources, and there is need to better match the magnitude of federal funding and response with the current state of the problem. Real-time data also provide a direct feedback loop by which the effectiveness of efforts can be more immediately assessed. Rates of overdose continue to increase and are at their highest levels ever experienced in the United States. Opioid overdose continues to be a defining public health challenge, and modernization of data and modeling approaches may help contribute to advances for this critical problem.

## Notes

Supplement. **eFigure.** Percent Error in Estimating National Opioid Overdose Fatality Rate by Number of Lagged Weeks of Data Used in LASSO Regression Models for Each Data Source, 2018 and 2019 **eAppendix.** Linear Regression Sensitivity Analyses: Results and Discussion
