# Supplementary material for: Estimating Weekly National Opioid Overdose Deaths in Near Real Time Using Multiple Proxy Data Sources
Source: JAMA Netw Open. 2022 Jul 21;5(7):e2223033. doi: 10.1001/jamanetworkopen.2022.23033 (PMC9305381; doi:10.1001/jamanetworkopen.2022.23033)
Supplement: Supplement. — eFigure. Percent Error in Estimating National Opioid Overdose Fatality Rate by Number of Lagged Weeks of Data Used in LASSO Regression Models for Each Data Source, 2018 and 2019 eAppendix. Linear Regression Sensitivity Analyses: Results and Discussion [file jamanetwopen-e2223033-s001.pdf]

Supplementary Online Content

Sumner SA, Bowen D, Holland K, et al. Estimating weekly national opioid overdose deaths in near real time using multiple proxy data sources. *JAMA Netw Open.* 2022;5(7):e2223033. doi:10.1001/jamanetworkopen.2022.23033

eFigure. Percent Error in Estimating National Opioid Overdose Fatality Rate by Number of Lagged Weeks of Data Used in LASSO Regression Models for Each Data Source, 2018 and 2019
eAppendix. Linear Regression Sensitivity Analyses: Results and Discussion

This supplementary material has been provided by the authors to give readers additional information about their work.

© 2022 Sumner SA et al. *JAMA Network Open.*

eFigure. Percent Error in Estimating National Opioid Overdose Fatality Rate by Number of Lagged Weeks of Data Used in LASSO Regression Models for Each Data Source, 2018 and 2019

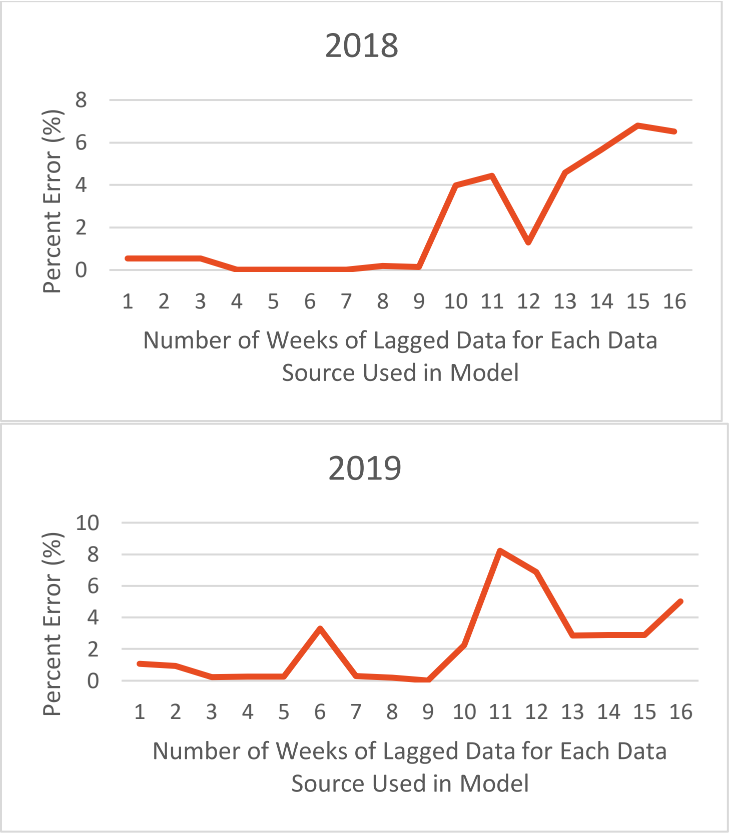

Note: Figure shows the absolute percent error for a Lasso regression model using each of the five data sources (National Syndromic Surveillance Program Emergency Department data; National Forensic Laboratory Information System reports; Google search trends; Twitter posts; and Reddit posts) by the number of lagged weeks used in the model. For example, results for 10 weeks of lagged data display the model performance when 10 weeks of lagged data are used for each of the 5 data sources (50 total predictor variables). Results show that, aside from a single small outlier at 6 weeks in 2019, percentage errors are low (around 1% or less) and relatively stable until approximately >9 weeks (2 months) of lagged data are used, at which point model errors increase and performance becomes more variable, likely as a result of model overfitting given the relatively small size of the dataset.

© 2022 Sumner SA et al. *JAMA Network Open.*

eAppendix. Linear Regression Sensitivity Analyses: Results and Discussion

We further examined whether the use of a Lasso regression model (a machine learning approach) held any benefit over a conventional ordinary least squares (OLS) linear regression, given the overall simplicity of the inputs to the model. The percentage error in the estimated annual opioid overdose fatality rate for 2018 (2.55%) and 2019 (-6.32%) revealed reasonable ability of an OLS model to generate estimates from proxy data sources; however, our empirical tests across two separate years indicate improved performance from a Lasso model. Our hypothesis for this improved performance is that, through model training and cross-validation on the training dataset, the Lasso model attempts to optimize the coefficients or relative weight assigned to each data source for predictive purposes. Conversely, an OLS regression forces all data sources to be considered in a model, even if not ideal for a particular year, and does not include model tuning through cross-validation on the training data set.

© 2022 Sumner SA et al. *JAMA Network Open.*
